# Knowledge of neonatal danger signs and associated factors among mothers who gave birth during the last 4 months while attending immunization services in Harar town public health facilities, Ethiopia, 2017

**DOI:** 10.1186/s13104-019-4677-2

**Published:** 2019-10-10

**Authors:** Fissaha Tekulu Welay, Nega Assefa Kassa, Gebrekiros Aregawi Gebremeskel, Natnael Etsay Assefa, Meresa Berwo Mengesha, Mulu Gebretsadik Weldemariam, Senait Gebreslasie Gebremeskel

**Affiliations:** 1 Department of Midwifery, College of Medicine and Health Science, Adigrat University, Adigrat, Ethiopia; 2 School of Public Health, College of Health and Medical Sciences, Haramaya University, Dire Dawa, Ethiopia; 3 Department of Midwifery, College of Medicine and Health Science, Aksum University, Aksum, Ethiopia

**Keywords:** Neonatal danger sign, Mothers’ knowledge, Immunization

## Abstract

**Objective:**

The purpose of this study was to assess knowledge about neonatal danger signs and associated factors among mothers who gave birth in the last 4 months attending immunization services.

**Result:**

The study recruited 432 mothers to participate. A knowledge score of neonatal danger signs was found [32.9% (95% CI 28.9%, 37%)]. Mothers educated to secondary level were 4.9 times more likely to know about neonatal danger signs [(AOR = 4.9, 95% CI (1.15, 21). Similarly, mothers whose husband educated to college and above [AOR = 4.95, 95% CI (1.15, 21)], and being multipara mother [(AOR = 2.59, 95% CI (1.05, 6.6)], were factors significantly associated with good knowledge of mothers about neonatal danger signs.

## Introduction

Parenthood of a new life is a responsibility for protection and crucial to the health and safety of the little, immune suppressed newborn. Although the symptoms of illness in a newborn baby are difficult to recognize, mothers always need to monitor their newborns for any signs of illness in the neonatal period. The majority of parents lack awareness about how a sick baby reacts as compared to a healthy baby. Thus, they lack experience in recognizing what is normal signs and what is unhealthy and atypical [1]. Neonatal danger signs that arose in the first month of life are multiple and can be a manifestation of almost any fatal diseases [2]. The integrated Management of Newborn and Childhood Illness (IMNCI), which is developed by the World Health Organization (WHO) introduces around ten general danger signs which define the illness of a neonate.

The critical period in newborn survival is often during the first week of life. During this period most neonatal deaths occur at home and without any contact with the formal health professional. Thus, unable to seek care and related problems are managed if mothers are aware of newborn danger signs through routine counseling by health professionals. Eventually, this will help them to develop the experience of early recognition. Hence, it is found necessary to assess mothers’ knowledge and associated factors that hinder and promote awareness of neonatal danger signs.

## Main text

### Methods

The quantitative institutional-based cross-sectional study design was applied to study a total of 433 mothers. The respondents were contacted at the baby immunization ward in different health facilities. Mothers with health problems like deafness and caregivers (Maidservant) who immunize their employer’s baby were excluded from the study to enhance the quality of data.

The required sample size was determined using a double population proportion formula by assuming a 95% level of confidence, a 5% margin of error. 80% power and the ratio of exposed to unexposed equivalent to 1. The highest number of samples was taken from the scenario at which mothers exposed to primary education to that of not taking formal education (18% vs. 8%) which yields 394. Thus, adding a 10% non-response rate, the total number of participants becomes 433 mothers. A total of six public health facilities (four health centers and two hospitals) were included in the study. Hence, a systematic sampling technique was used from their registration frame to get a total of 433 participants after proportionally allocate to size for each health facility.

A structured, questionnaire developed by the principal investigator was applied in this study. The questionnaire was first prepared in the English language and translated to local languages for the ease of the interview. It was also pretested on 5% of the population out of the study area. Finally, the mothers were interviewed face- to- face using the checked and pretested questionnaires.

#### Operational definition

##### Knowledge

A mother who mentions three of the ten WHO recognized danger signs of a neonate without prior prompt, plus three or more danger signs with a prior prompt is categorized as good knowledge. But, mothers capable of mentioning two or fewer key danger signs of neonate with and without prior prompt are classified as having poor knowledge [3].

The collected data were entered into epi data and exported to SPSS version 20 for analysis. All variables with p-value ≤ 0.2, on bivariate logistic regression analysis, were taken into the multivariate model to control the possible confounders. The odds ratio (OR) was used as a measure of strength and the level of statistical significance was declared at p-value < 0.05.

Finally, before the data collection process started, ethical clearance was secured from Haramaya University Institutional Health Research Ethics Review Committee (IHRERC). Official letter was disseminated from Haramaya University College of health and medical sciences to each selected health facilities.

### Result

#### Socio-demographic characteristics

A total of 432 mothers of babies aged up to 4 months were included in the study. The median age of the mothers was 25 years with a range of 15–45 years (Additional file 1: Table S1).

#### Knowledge of neonatal danger signs

**Fig. 1:**
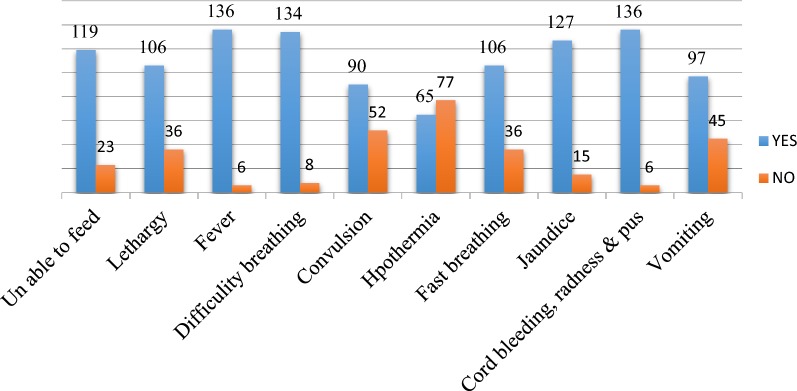
Knowledge of prompted neonatal danger signs among mothers who gave birth the last 4 months attending immunization in Harar town public health facilities, Eastern Ethiopia, February 2017

Out of the 432 recruited mothers, 302 (69.9%) mothers were aware of the following number of mentioned neonatal dangers sign, one, 60 (13.9%), two, 100 (23.1%) and 142 (32.9%) three and above. Mothers who satisfied the WHO criteria for good knowledge was found 142 (32.9% with 95% CI 28.9%, 37%). Among the prompted neonatal danger signs cord bleeding, redness followed by pus, and fever mentioned by 95.8%, while Hypothermia, convulsion, and vomiting are the least mentioned neonatal danger signs accounted for 45.8%, 63.4%, and 68.3% respectively (Fig. 1).

#### Maternal health service utilization and obstetric conditions

Out of 432 mothers, 393 (90.9%) had antenatal care follow up (ANC) during the latest pregnancy. Counseling related to neonatal danger signs were the least of all area of counseling covered with 34.2%. Similarly, out of 432 mothers less than half, 133 (30.8%) had postnatal care visits (PNC) and only 70 (52.6) mothers got counseled about neonatal danger signs (Additional file 1: Table S2).

The median parity of mothers was two (2), ranging from 1 to 8 live births with multipara mothers 226 (52.3%) accounted for the highest number in the parous status of the respondents. Related to the place of delivery the majority of mothers 352 (81.5%) delivered their current baby in a hospital for labor and delivery (Additional file 1: Figure S1).

#### Source of information

**Fig. 2:**
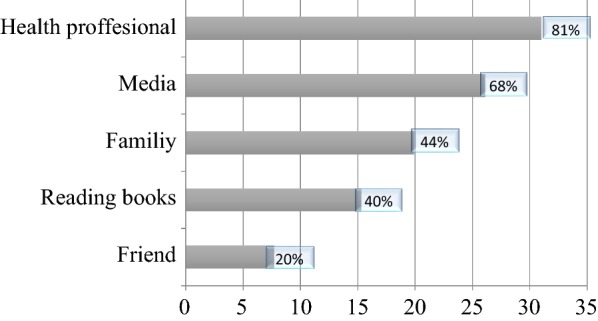
First source of information about neonatal danger signs for mothers who gave birth the last 4 month attending baby immunization in Harar town public health facilities, Eastern Ethiopia, February 2017 (n = 253)

Regarding the source of information 253 (83.8%) mothers from those who were aware of neonatal danger signs had been informed from different sources. Among this subset, 81 (31%) had received information from health professionals (Fig. 2).

#### Mother’s newborn care knowledge and practice

The majority, 280 (64.8%) mothers knew cord care. Regarding cord care practice, 218 (78.2%) but nothing on the cord, 42 (14.6%) butter, and 18 (6.4%) other remedies. Concerning the mother’s neonatal care practice, about 306 (70.8%) reported as the exact time of breastfeeding initiation is within 1 h, while less than half 153 (35.4%) mothers claimed as the exact time of baby bath is within 24 h. delivery and 320 (74.1%) mothers were reported as they use an insecticide-treated net for their newborn baby.

#### Factors associated with knowledge of neonatal danger signs

The multi-variable analysis result showed that educated mothers to the level of college and above as well as secondary level were more likely to recognize neonatal danger signs (Table 1).

**Table 1:** Factors associated with knowledge of neonatal danger signs among mothers who gave birth the last 4 month attending immunization in Harar town public health facilities, Eastern Ethiopia, February 2017

Independent variables	Frequency (%)	Knowledge of neonatal danger signs		COR (95% CI)	AOR (95% CI)
		Good	Poor		
Maternal level of education					
College and above	95 (27.3)	53	42	3.99 (1.86, 8.6)**	5.4 (1.3, 22.7)*
Secondary level	83 (23.9)	38	45	2.7 (1.22, 5.83)*	4.9 (1.15, 21.1)
No formal education	50 (14.4)	12	38	1.00	1.00
Time of breast feeding initiation					
< 1 h	306 (70.8	113	193	1.87 (1.17, 3)**	3.23 (1.11-9.7)
> 1 h	126 (29.2)	30	96	1.00	1.00
Husband/partner level of education					
College and above	88 (20.6)	44	44	3.4 (1.72, 6.75)**	4.95 (1.15, 21.32)**
Secondary level	106 (24.8)	43	63	2.34 (1.2, 4.53)*	4.7 (1.15, 19.23)
No formal education	75 (17.5)	17	58	1.00	1.00
Parity					
Primi para	188 (43.5)	46	142	1.00	1.00
Multi para	226 (52.3)	85	141	1.9 (1.2, 2.9)**	2.59 (1.05, 6.58)*
ANC neonatal danger sign counseling					
Yes	121 (34.2)	76	45	4.87 (3.04, 7.8)**	4.34 (1.61, 11.7)
No	233 (68.8)	60	173	1.00	1.00
PNC visit within 6 days					
Yes	145 (33.6)	74	71	3.29 (2.16, 5.02)**	3.32 (1.26, 8.67)
No	287 (66.4)	69	218	1.00	
Source of information					
Yes	253 (83.8)	127	126	2.29 (1.19, 4.4)*	6.04 (1.63, 22.4)**
No	49 (16.2)	15	34	1.00	1.00
Mode of delivery					
Spontaneous vaginal delivery (SVD)	248 (57.4)	51	197	1.00	1.00
Caesarian section	148 (34.2)	80	68	4.5 (2.9, 7)**	3.86 (1.46, 10.25)
First source of information					
Family	52 (19.9)	12	40		
Health professional	81 (31)	47	34	4.6 (2.1, 10.6)**	5.45 (1.27, 23.3)*
Media	68 (26.1)	35	33	3.5 (1.6, 7.9)*	5.08 (1.16, 22.36)*

*CI* Confidence Interval, *COR* Crude Odds Ratio, *AOR* Adjusted Odds Ratio

^*^p-value < 0.05, ^**^p-value < 0.001

### Discussion

In this study, the knowledge of neonatal danger signs was found to be 32.9% (95% CI 28.9%, 37%), 37%). It was found satisfactory on educated mothers and those who got ANC counseling about neonatal danger signs at their current ANC follow up visit. The knowledge of neonatal danger signs in this study was lower than the study done in India (2006), which was 39% [4], in Nigeria (2009) which was 78.3% [5], in Egypt (2008) which was 69% [6] and in Tigray region Ayder referral hospital (2011), 64% [7]. Even though, the most frequently mentioned danger sign was fever, in all four studies, which is congruent with this study. The knowledge gap may be due to socioeconomic differences lead to owning an advanced health care delivery system. According to this study, mothers who were educated to a secondary level and college and above were more likely to know about neonatal danger signs compared to those who didn’t take formal education. This was nearly consistent with the study conducted in Gondar, (2012) that mothers with the same level of education were more prone to know ≥ 3 neonatal danger signs with and without prior prompt [8]. This might be because of education increases the tendency to get service and read materials related to their baby.

The study revealed that mothers who were counseled about neonatal danger signs during their current ANC follow up and those who had PNC visits during the first 6 days postpartum were more likely to have good knowledge about neonatal danger signs. Thus, it is in line with the study conducted in Fiche town Oromia region in 2012 [9], and in Gondar, 2012 [8]. The reason might be, increased maternal ANC and PNC utilization promote the likelihood of counseling and knowledge acquisition. In our study, we tried to test new variables; among them, a mode of delivery was found to be significantly associated. Mothers who delivered through cesarean section were found more knowledgeable about neonatal danger signs compared to those who delivered by spontaneous vaginal delivery (SVD). This might be the longer they stay at a health facility in the postnatal period, the more to get counseled about neonatal danger signs.

Mothers who had a source of information about were six times more to know the danger signs compared to those who didn’t have. In line with that, mothers who got information from health professionals and media were more knowledgeable about neonatal danger signs. The finding is similar with the study conducted in India by 2012, where poor knowledge was due to other sources than health professional [10] and with the study conducted in Gondar (2012), by which mothers got their first information about neonatal danger sign from health professionals and media have significantly resulted good knowledge [8].

Mothers who knew the exact breastfeeding initiation, as less than 1 h (78%) in our study were found more knowledgeable compared to those who reported as later than 1 h. This is consistent with the study in Nepal, Chitwan district (2011) even though the number of mothers who reported as less than 1 h differs (78% vs. 52%) [11], the similarity might, due to more emphasis is given by the government upon counseling breastfeeding habit of mothers, which increases bonding and identifying the possible newborn illness.

## Limitation

- Because this study used a cross-sectional study design, it is descriptive and cannot demonstrate a specific relationship between cause and effect.
- The inclusion of mothers up to 4-month post-partum may result in some recall bias given that infant health concerns and danger signs are common during the first month of life.

## Supplementary information

**Additional file 1: Table S1.** Sociodemographic characteristics of mothers who gave birth the last 4 months attending baby immunization in Harar town public health facilities, Eastern Ethiopia, February 2017. **Table S2.** Antenatal care service utilization and obstetric conditions of mothers who gave birth the last 4 month attending immunization in Harar town public health facilities, Eastern Ethiopia, February 2017. **Figure S1.** Place of delivery of mothers who gave birth the last 4 month attending baby immunization in Harar town public health facilities, Eastern Ethiopia, February 2017 (n = 432)

## Data Availability

The datasets during and/or analyzed during the current study available from the corresponding author on reasonable request.

## Abbreviations

ANC: antenatal care
AOR: adjusted odd ratio
BCG: Bacillus Chalmette Guerin
CI: confidence interval
HEWs: Health Extension Workers
HC: health center
HU-IHRERC: Haramaya university institutional health research ethics review committee
IMNCI: integrated management of newborn and childhood illness
OR: odds ratio
PI: Principal Investigator
PNC: postnatal care
SNNP: southern nation nationalities and peoples of Ethiopia
SPSS: statistical package for social science
SVD: spontaneous vaginal delivery
UNICEF: United Nation Children’s Emergency Fund
WHO: World Health Organization
