# Supplementary material for: Knowledge of neonatal danger signs and associated factors among mothers who gave birth during the last 4 months while attending immunization services in Harar town public health facilities, Ethiopia, 2017
Source: BMC Res Notes. 2019 Oct 10;12:651. doi: 10.1186/s13104-019-4677-2 (PMC6785929; doi:10.1186/s13104-019-4677-2)
Supplement: Supplementary file 1 — Additional file 1: Table S1. Sociodemographic characteristics of mothers who gave birth the last 4 months attending baby immunization in Harar town public health facilities, Eastern Ethiopia, February 2017. Table S2. Antenatal care service utilization and obstetric conditions of mothers who gave birth the last 4 month attending immunization in Harar town public health facilities, Eastern Ethiopia, February 2017. Figure S1. Place of delivery of mothers who gave birth the last 4 month attending baby immunization in Harar town public health facilities, Eastern Ethiopia, February 2017 (n = 432) [file 13104_2019_4677_MOESM1_ESM.docx]

Table S1. Sociodemographic characteristics of mothers who gave birth the last 4 months attending baby immunization in Harar town public health facilities, Eastern Ethiopia, February 2017

| Variables | | Frequency | Percentage |
| --- | --- | --- | --- |
| Maternal religion(n=432) | Muslim  Orthodox  Catholic  Protestants | 206  188  4  34 | 47.2  43.5  0.9  7.9 |
| Maternal level of education  (n=348) | No formal education | 50 | 14.4 |
|  | Primary level | 120 | 34.5 |
|  | Secondary level | 83 | 23.9 |
|  | College and above | 95 | 27.3 |
| Maternal marital status  (n=432) | Married/partner | 427 | 98.8 |
|  | Divorced/separated | 5 | 1.2 |
| Husband educational status  (n=428) | No formal education | 75 | 17.5 |
|  | Primary level | 159 | 37.1 |
|  | Secondary level | 106 | 24.8 |
|  | College and above | 88 | 20.6 |
| Maternal age(n=432) | <18 year  18-31 year  >31 year | 7  381  44 | 1.6  88.2  10.2 |
| Maternal residence(n=432) | Urban | 408 | 94.4 |
|  | Rural | 24 | 5.6 |

Table S2. Antenatal care service utilization and obstetric conditions of mothers who gave birth the last four month attending immunization in Harar town public health facilities, Eastern Ethiopia, February 2017

| Variable | |  |  | |  |  | | Frequency | | |  | | Percentage | | | |  |
| --- | --- | --- | --- | --- | --- | --- | --- | --- | --- | --- | --- | --- | --- | --- | --- | --- | --- |
| Recent ANC follow up  (n= 432) |  |  | | Yes  (n=393) |  | | One | | 42 | | | | |  | 10.7 | |  |
|  |  |  |  |  |  |  | Two | | 94 | | | | |  | 23.9 | |  |
|  |  |  |  |  |  |  | Three | | 96 | | | | |  | 24.4 | |  |
|  |  |  |  |  |  |  | Four and above | | 161 | | | | |  | 41 | |  |
|  |  |  | | No  (n=39) |  | | | | |  | | | | 9 | |  |  |
| Parity  (n=432) |  | Primi para (1) | | | | | | | 188 | | |  | | 43.5 | | | |
|  |  | Multi para (2-4) | | | | | | | 226 | | |  | | 52.3 | |  |  |
|  |  | Grand multi para (≥5) | | | | | | | 19 | | |  | | 4.2 | |  |  |
| Mode of delivery  (n=432) |  | Spontaneous vaginal delivery | | | | | | | 248 | | |  | | 43.5 | |  |  |
|  |  | Instrumental delivery | | | | | | | 36 | | |  | | 8.3 | |  |  |
|  |  | Caesarian section | | | | | | | 148 | | |  | | 34.2 | |  |  |


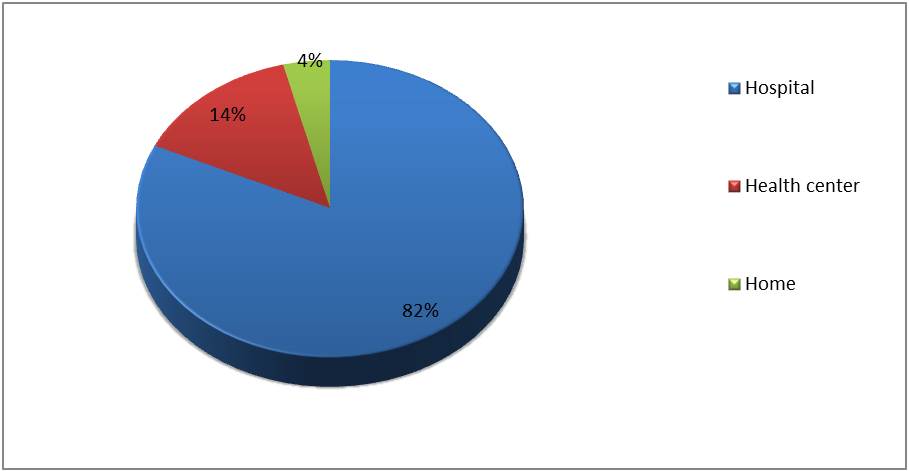


Figure S 1. Place of delivery of mothers who gave birth the last four month attending baby immunization in Harar town public health facilities, Eastern Ethiopia, February 2017(n=432)
